# Risk factors associated with posttraumatic stress disorder in US veterans: A cohort study

**DOI:** 10.1371/journal.pone.0181647

**Published:** 2017-07-25

**Authors:** Jan Müller, Sarmila Ganeshamoorthy, Jonathan Myers

**Affiliations:** 1 Institute of Preventive Pediatrics, Technische Universität München, Munich, Germany; 2 Division of Cardiology, Veterans Affairs Palo Alto Health Care System, Palo Alto, California, United States of America; 3 Stanford University School of Medicine, Stanford, California, United States of America; Stellenbosch University, SOUTH AFRICA

## Abstract

**Objective:**

To assess the association between clinical and exercise test factors and the development of posttraumatic stress disorder (PTSD) in US Veterans.

**Patients and methods:**

Exercise capacity, demographics and clinical variables were assessed in 5826 veterans (mean age 59.4 ± 11.5 years) from the Veterans Affairs Healthcare System in Palo Alto, CA. The study participants underwent routine clinical exercise testing between the years 1987 and 2011. The study end point was the development of PTSD.

**Results:**

A total of 723 (12.9%) veterans were diagnosed with PTSD after a mean follow-up of 9.6 ± 5.6 years. Drug abuse (HR: 1.98, CI: 1.33–2.92, p = .001), current smoking (HR: 1.57, CI: 1.35–2.24, p <.001), alcohol abuse (HR: 1.58, CI: 1.12–2.24, p = .009), history of chest pain (HR: 1.48, CI: 1.25–1.75, p <.001) and higher exercise capacity (HR: 1.03, CI: 1.01–1.05, p = .003) were strong independent risk factors for PTSD in a univariate model. Physical activity pattern was not associated with PTSD in either the univariate or multivariate models. In the final multivariate model, current smoking (HR: 1.30, CI: 1.10–1.53, p = .002) history of chest pain (HR: 1.37, CI: 1.15–1.63, p <.001) and younger age (HR: 0.97, CI: 0.97–0.98, p <.001) were significantly associated to PTSD.

**Conclusions:**

Onset of PTSD is significantly associated with current smoking, history of chest pain and younger age. Screening veterans with multiple risk factors for symptoms of PTSD should therefore be taken into account.

## Introduction

Posttraumatic stress disorder (PTSD) is a psychiatric condition that develops after experiencing or witnessing a traumatic event such as warfare, sexual assaults, natural disasters, or other life threatening events [1–3]. It is associated with persistent mental and emotional stress, substance abuse, smoking, increased violence, poor work performance and poor quality of life in US war veterans [3–5]. The National Vietnam Veterans Readjustment Study reported a lifetime incidence of PTSD of 30% and a current prevalence of 15%, whereas the normal estimated lifetime occurrence of PTSD is 6.8% [2, 6].

The association between history of smoking, substance abuse and alcohol abuse in posttraumatic stress disorder have been well established [7–10]. Chronic substance use can increase anxiety and agitation. In an event of traumatic exposure, substance use affects the neurological systems thus increasing the vulnerability of developing PTSD [11]. Therefore, PTSD is considered a causal risk factor for substance use disorders, when substances are used to relieve distressing symptoms of PTSD [3, 9].

Individuals with PTSD also have a high prevalence of comorbidities associated with low physical activity, poor fitness or both, including obesity, diabetes and metabolic syndrome [12–14]. It has been demonstrated that physical activity is effective in decreasing depressive symptoms associated with PTSD [14] and may also be useful for improving other health conditions among individuals with PTSD including exercise capacity, and components of the metabolic syndrome such as waist circumference, blood pressure and blood lipid levels which increase cardiovascular risk [13].

The Veterans Exercise Testing Study (VETS), is an ongoing, prospective evaluation of Veteran subjects referred for exercise testing for clinical reasons, designed to address exercise test, clinical, and lifestyle factors and their association with health outcomes. Given that Veterans have a particularly high prevalence of PTSD, the VETS cohort provided an opportunity to assess the prevalence and factors, particularly exercise capacity and physical activity patterns, which might influence the development of PTSD.

## Patients and methods

A cohort of 5826 Veterans (59.4 ± 11.5 years) who were referred for a single maximal treadmill test at the Veterans Affairs Palo Alto Health Care System between 1987 and 2011 were considered. 335 Patients with PTSD disorder before the exercise test were excluded from the study. Historical information that was recorded at the time of the exercise test included previous myocardial infarction by history or presence of Q-waves, cardiac procedures, heart failure, hypertension, hypercholesterolemia (>220 mg/dL, statin use, or both), claudication, chronic obstructive pulmonary disease, cancer, renal disease, diabetes, stroke, smoking status (never, former, and current smoker), and use of cardiac medications. Diabetes status was classified as use of insulin or oral hypoglycemic agents. Participation in regular activity was evaluated by a patient’s binary response to the question: “At least 3 times a week, do you engage in some form of regular activity such as brisk walking, jogging, bicycling, or swimming, long enough to work up a sweat, get your heart thumping, or become short of breath?” Subjects who answered yes to this question were classified as meeting the minimal criteria for physical activity outlined by the American College of Sports Medicine (ACSM) Guidelines [15, 16]. The study was approved by the Investigational Review Board (IRB) at Stanford University (Board number: 12061)

### Exercise testing

Participants underwent symptom limited treadmill testing using an individualized ramp treadmill protocol as described previously [17]. Standard criteria for termination were used including moderately-severe angina, >2.0 mm horizontal or down sloping ST segment depression, decrease in systolic blood pressure and serious rhythm disturbances. The Borg 6–20 perceived exertion scale was used to quantify degree of effort. Blood pressure was taken manually, and exercise capacity (in metabolic equivalents [METs]) was estimated from peak treadmill peak speed and grade (ACSM). Medications were not withheld. Subjects whose tests were terminated prematurely because of orthopedic or other limitations were excluded.

### Outcomes

The study end point was the development of PTSD (ICD-9-CM 309.81) as diagnosed and recorded electronically by the patient’s primary care physician. The Veterans Affairs computerized medical records system (CPRS) was used to verify date of onset of PTSD.

### Statistical analysis

All statistical analyses were preformed using SPSS software (IBM SPSS Statistics for Windows, Version 23.0). Descriptive data are expressed as mean ± standard deviation and categorical variables are presented in absolute numbers or as percentages where appropriate. Comparisons of patients developing vs. not developing PTSD were performed using unpaired student’s t-tests for continuous variables and chi^2^ tests for categorical variables.

Independent associations with onset of PTSD were estimated using Cox proportional hazards analysis. Two separate models were developed. First, the effect of anthropometrics, cardiovascular and other risk factors, exercise and medications were assessed in a univariate model. Second, all variables that exhibited a significant univariate association were tested in a multivariate model. Receiver Operating Characteristic (ROC) curves were used to derive the cut-off-value for highest sensitivity and specify from the multivariate model. Kaplan-Meier survival curves were generated to illustrate the association between risk factors including smoking, chest pain and age and the onset of PTSD. For all analyses, a probability value of p<0.05 was considered statistically significant.

## Results

In total, 723 (12.9%) patients were diagnosed with PTSD after a mean follow-up of 9.6 ± 5.6 years. Table 1 summarizes patients’ characteristics including demographics, risk factors and medications among the entire study group, and in patients who developed and did not develop PTSD.

**Table 1:** Patients characteristic.

	Whole Study Group **(n = 5826)**	No PTSD **(n = 5076)**	PTSD **(n = 750)**	p-value*
**Gender (male)**	5619	4896	723	.916
**Age (years)**	59.4 ± 11.5	60.1 ± 11.6	54.9 ± 11.5	**<.001**
**Follow-up (years)**	9.6 ± 5.6	10.1 ± 5.6	6.1 ± 5.0	**<.001**
**BMI (kg/m**^**2**^**)**	29.0 ± 5.3	28.9 ± 5.3	29.2 ± 5.4	.152
**Exercise Capacity (MET)**	8.24 ± 3.4	8.16 ± 3.5	8.82 ± 3.3	**<.001**
**Risk Factors**				
**History of CVD**	1409 (24.2%)	1270 (25.0%)	139 (18.5%)	**.001**
**History of Hypertension**	3133 (53.8%)	2756 (54.2%)	377 (50.3%)	**.041**
**History of Dyslipidemia**	2194 (37.7%)	1904 (37.5%)	290 (38.7%)	.545
**History of Drugs**	135 (2.3%)	109 (2.1%)	26 (3.5%)	**.036**
**History of Alcohol Abuse**	228 (3.9%)	194 (3.8%)	34 (4.5%)	.363
**History of Diabetes**	955 (16.4%)	837 (16.5%)	118 (15.7%)	.635
**Current Smoking**	1438 (24.7%)	1189 (23.4%)	249 (33.2%)	**<.001**
**Physical Inactive**	2595 (44.5%)	2275 (44.8%)	320 (42.7%)	.271
**Medication**				
**Beta-blocker**	1287(22.1%)	1143 (22.5%)	144 (19.2%)	.**043**
**ACE Inhibitor**	1380 (23.7%)	1215 (23.9%)	165 (22%)	.250
**Antihypertensive**	954 (16.4%)	859 (16.9%)	95 (12.7%)	**.003**
**Diuretics**	397 (6.8%)	350 (6.9%)	47 (6.3%)	.587
**Statins**	819 (14.1%)	730 (14.4%)	89 (11.6%)	.072

BMI: Body Mass Index, MET: metabolic equivalent, CI: confidence interval, ACE: Angiotensin-converting enzyme, CVD: Cardiovascular disease

*comparing patients with cognitive impairment to those without by a Student’s t-test or chi^2^ if appropriate

As shown in Table 2, drug abuse (HR: 1.98, CI: 1.33–2.92, p = .001), current smoking (HR: 1.57, CI: 1.35–2.24, p <.001), history of alcohol abuse (HR: 1.58, CI: 1.12–2.24, p = .009), history of chest pain (HR: 1.48, CI: 1.25–1.75, p <.001) and higher exercise capacity (HR [per MET]: 1.03, CI: 1.01–1.05, p = .003) were significant independent risk factors for PTSD in a univariate model. Higher age (HR: 0.970, Cl: 0.964–0.977, p<0.001), use of antihypertensive drugs (HR: 0.735, Cl: 0.592–0.911, p = 0.005) and history of cardiovascular disease (HR: 0.751, Cl: 0.624–0.902, p = 0.002) made PTSD more unlikely in the univariate model (Table 2). Physical activity pattern were not associated with PTSD.

**Table 2:** Univariate and multivariate Cox proportional hazard analysis which were independently associated to PTSD in 5826 veterans.

	Bivariate Model		Multivariate Model	
	Hazard Ratio (95% CI)	p-value	Hazard Ratio (95% CI)	p-value
**Age (years)**	0.970 (0.964–0.977)	**<.001**	0.973 (0.966–0.981)	**<.001**
**BMI (kg/m**^**2**^**)**	1.007 (0.995–1.021)	.259	-	-
**Exercise Capacity (MET)**	1.028 (1.010–1.047)	**.003**	0.995 (0.970–1.021)	.699
**History of CVD**	0.751 (0.624–0.902)	**.002**		
**History of Drugs**	1.975 (1.334–2.924)	**.001**	0.863 (0.709–1.050)	.141
**History of Alcohol Abuse**	1.586 (1.123–2.240)	**.009**	1.138 (0.749–1.728)	.544
**History of Chest Pain**	1.475 (1.247–1.745)	**<.001**	1.366 (1.147–1.626)	**<.001**
**Current Smoking**	1.574 (1.352–1.832)	**<.001**	1.297 (1.101–1.529)	**.002**
**Physical Inactive**	1.075 (0.931–1.243)	.325	-	-
**Beta-blocker**	0.903 (0.753–1.083)	.272	-	-
**ACE Inhibitor**	1.072 (0.901–1.275)	.443	-	-
**Antihypertensive**	0.735 (0.592–0.911)	**.005**	0.824 (0.661–1.028)	.086
**Diuretics**	1.203 (0.894–1.618)	.226	-	-
**Statins**	1.020 (0.816–1.276)	.862	-	-

BMI: Body Mass Index, MET: metabolic equivalent, CI: confidence interval, ACE: Angiotensin-converting enzyme, CVD: Cardiovascular disease

**Fig 1:**
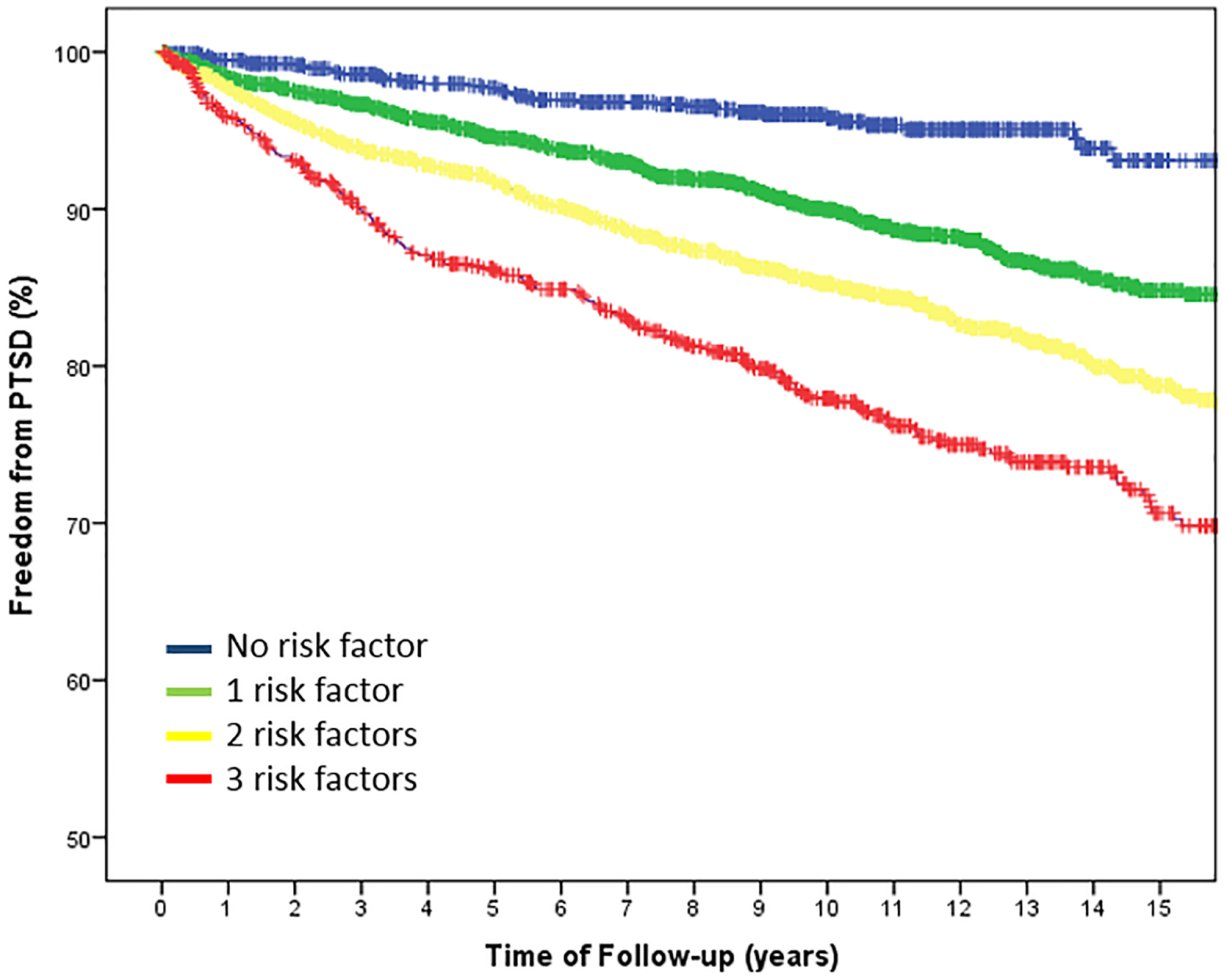
Kaplan-Meier charts for Freedom from PTSD stratified for the three independent risk factors smoking, chest pain and younger age.

In the final multivariate model (Table 2) only current smoking (HR: 1.30, CI: 1.10–1.53, p = .002) history of chest pain (HR: 1.37, CI: 1.15–1.63, p <.001) and younger age (HR: 0.97, CI: 0.97–0.98, p <.001) remained significantly associated with PTSD. ROC analysis revealed that an age <59.4 years had the highest sensitivity and specificity for onset of PTSD. Freedom from PTSD stratified for the three independent risk factors current smoking, history of chest pain and age younger than 59.4 years is illustrated in Fig 1.

## Discussion

This study shows that younger age, current smoking and history of chest pain were strong independent risk factors for developing PTSD. PTSD was less prevalent in older than younger veterans. Different perception of symptoms including higher somatic complaints and lower arousal, guilt or depression are reported in older veterans. Selective mortality may also contribute to these findings since it has been shown that veterans with more severe symptoms die younger [18]. An even simpler explanation might be that PTSD is a “younger” disease and is therefore just overlooked in the older population [19]

Our results suggest a possible link between substance abuse and development of PTSD given that history of drug and alcohol abuse were significantly associated with PTSD in the univariate model. This may be attributable to the tendency for those with PTSD to abuse drugs in order to improve PTSD symptoms. Substance abuse decreases concentration and is associated with irritability, insomnia, aggression and anger. Substance abuse may also increase hyperarousal and re-experience of PTSD symptoms as well as represent a form of self-medication [1, 11]. Other studies have shown that CNS depressants such as alcohol and drugs acutely improve PTSD symptoms[10]. Furthermore, the severity and duration of substance abuse accelerates symptoms of PTSD [11, 20]. Vujanovic et al. reported that individuals with cocaine-use disorders, compared to those without, had a two-fold higher incidence of PTSD [1]. Chronic substance abuse can lead to higher levels of arousal which makes an individual more vulnerable to the development of PTSD [11]. Studies of PTSD patients with cocaine dependence suggest that cocaine users were more likely to suffer from comorbid major depression than were patients in whom PTSD developed after the onset of cocaine use [10]. Depression changes mood, decreases concentration and causes insomnia. These symptoms may contribute to hyperarousal thus contributing to development of PTSD.

In the present study, drug and alcohol abuse were univariately related to PTSD but only current smoking remained in the multivariate model. Other reports suggest that patients with PTSD use cigarettes to reduce the negative effects that arise from daily stressors [21]. Beckham et al. examined smoking in subjects with PTSD and observed that motivation to smoke cigarettes was attributable to an effort to reduce negative affect [8]. In response to trauma and stress, smokers with PTSD reported greater increases in negative affect and cigarette craving compared to smokers without PTSD. In addition, those with PTSD were more likely to smoke in response to negative affect and were more likely to report a reduction in negative affect after smoking a cigarette than those without PTSD [22].

A mutual maintenance theory model has been developed in order to explain the complex interaction between PTSD and pain [23]. In our study chest pain was an important contributor to the development of PTSD; a 31% increase in the development of PTSD was observed among Veterans with a history of chest pain. Chest pain may be related to re-experiencing symptoms of PTSD and may serve as symbolic or actual reminder of their traumatic exposures. The pain may be due to stressors or related to other psychiatric disorders such as anxiety and depression and somatic symptoms in PTSD patients [24, 25]. Reduced levels of activity are also common in both chronic pain and PTSD leading to increased levels of disability [23]. Anxiety in PTSD patients may directly influence the perception of pain [25–27]. In PTSD, aggravation of pain or maintenance of pain might be due to efforts made to control the pain by behavioral avoidance and physiological arousal [27, 28]. Future studies should be conducted to determine underlying causes of chest pain and related distress in chronic PTSD patients. Other studies should be designed to determine whether chest pain is associated with hypochondriasis, hysteria or re-experiencing symptoms among combat Veterans with PTSD and the extent to which chest pain causes disability.

Physical activity that goes hand in hand with physical fitness is often recommended as a treatment for psychiatric disorders [14]. In the univariate model we observed contrasting results given that for every MET increase the risk of developing PTSD increased by 3%. We can assume that this is due to bias in data collection. Among PTSD patients, exercise levels are often lower due to their depressive mood [29]. In addition, physical fitness might be associated or confounded by history of cardiovascular disease and antihypertensive treatment that showed only bivariate associations. It should be noted that hypertension increases the risk of intellectual dysfunction by increasing susceptibility to ischemic brain injury and cerebrovascular pathology causing cognitive decline. Therefore antihypertensive agents are used as protective mechanisms. For example, Prazosin is a classic example of antihypertensive medication that is commonly used to treat the trauma-related nightmares in PTSD. A complete understanding of the interactions between these cardiovascular risk factors, medications, drug abuse, alcohol and smoking is elusive and requires further study.

Since exercise is associated with other health benefits such as a reduction of cardiovascular risk factors, it might also help patients to develop a more optimistic view of their overall health. In our study there was neither an association between PTSD incidence and exercise capacity or physical activity in the multivariate model. Possible reasons might be the single question item to assess physical activity and that both physical activity and fitness were only assessed once and might have changed over time. Nevertheless, various types of physical activity interventions, including aerobic exercise or strength and flexibility training, significantly decrease clinical symptoms of mood and anxiety disorders [30, 31]. Bandura et al. [30] proposed social cognitive theory to modulate behavioral changes. Physical activity may interrupt the vicious cycle of pain, fear, and mood disorders. In addition, Manger and Motta's aerobic exercise intervention for PTSD showed promising results for symptom management, including reductions in depression and anxiety symptoms [32].

## Conclusion

PTSD is a highly prevalent lifetime disorder common in the military service. Current smoking, history of chest pain and younger age were significantly related to the onset of PTSD. Although physical activity and fitness were not associated with development of PTSD, exercise has been demonstrated to be an effective intervention in treating patients with PTSD and should be included in the rehabilitation process. Identifying further risk factors for the development of PTSD is important for understanding and developing better treatment strategies for this disorder.

## Limitations

Reverse causality, which is the possibility that there may have been patients with subclinical PTSD at the time of the exercise test that influenced the outcome cannot be ruled out. The operationalization of PTSD onset is difficult because many factors influence medical record entries. Occurrence rate of drug and alcohol abuse was low, which may have limited their significance in the multivariate model. Physical activity was assessed by only a single question which might explain its lack of association with PTSD. Our study sample was comprised entirely of male subjects and the results may not be applicable to women. Several risk factors including physical inactivity, smoking, drug or alcohol abuse as well as cardiovascular history were based on recall, and therefore may not have been defined precisely. Finally, all baseline characteristics were only assessed once and may have changed over time.

## Supporting information

S1 File Original dataset. (XLS)
